# Transactions of Societies

**Published:** 1828-04-01

**Authors:** 


### TRANSACTIONS OF SOCIETIES.

### 1. ANEURISM.

In a late sitting of the Westminster Medical Society, Dr. Barry furnished a subject for discussion—namely, that of *internal* aneurism. The reason for the *internal* consideration of the subject was humourously enough grounded on the distinction between physic and surgery. Thus, till an aneurism points above the clavicle or below Poupart's ligament, it is, of course, the property of the physician—but the moment it overleaps these boundaries, the surgeon claims it as his prescriptive right! True it is, that the surgeons *sometimes* invade the domain of the physician. Cooper and Abernethy ought to be prosecuted and fined by the College of Physicians for digging into the abdomen—a soil in which they had no right to plunge a spade; while Wardrop and many others have passed the line of demarcation, in an opposite direction, and have theatened to tie up the ventricles or auricles of the heart itself, when affected with aneurism! We have a plea for canvassing the opinions and practices of *both* parties—because, like Tyresias, of old, we have served in both capacities.

If aneurism were as common in ancient as in modern times, it is wonderful that it should have escaped the observation of Hippocrates and Celsus. The latter clearly understood the difference between veins and arteries, and observed that wounds of the latter would not heal. "At arteria incisa neque coit, neque sanescit; interdum etiam, ut sanguis vehementer erumpat, efficet."—*Lib.* ii. But the word aneurism does not occur in his writings. He describes *varices* of the veins of the leg, and directs them to be cut out, having first placed a ligature above and below the tumour. In wounds of the veins, too, he directs a ligature above and one below the wound, and then the vessel to be entirely cut across. "*Venæ*, quæ sanguinem fundunt apprehendendæ, circaque id quod ictum est, duobus locis delegandæ, intercedendæque sunt."—*Lib.* v. It must not be said that Celsus here means an artery when he speaks of a vein; for he has minutely described the differences between these vessels, not only in their coats, but in the kinds of blood which they contain. If this opinion be doubted, we have the authority of Sprengel on our side. Ætius seems to be the first who distinctly notices aneurism, when speaking of Philagrius. He says that that bold surgeon excised the aneurismal tumour in toto, having first tied the artery above and below. Paulus Eginetus, and almost all the surgeons of the middle ages, followed the above method; which afterwards fell into disuse. Lanfranc was the first to recommend the actual cautery to the aneurismal opening in the artery, which advice was adopted by Fallopius. Marcus Aurelius Severinus appears to have been the first who tied the femoral artery close to Poupart's ligament, for an aneurism at the top of the thigh.* Thus, till the sixteenth century, the treatment of aneurism was by ligature—and, in some rare cases, by excision of the tumour. At this epoch, John of Vigo tried compression and styptics, which were employed by Ambrose Paré, Fabricius Hildanus, &c. About the middle of the seventeenth century, the tourniquet was employed by some surgeons, to check the flow of blood into the aneurismal sac, while various contrivances were adopted to make pressure on the tumour itself. Passing over these, it appears that Dominique Anel was the first to propose, and Lacorta to perform the operation of tying the artery both above and below the aneurism, without making any opening into the latter. Then came the operation of John Hunter, so well known, and still so universally adopted. But we cannot always, as in popliteal aneurism, tie the artery at a distance above the disease—nay, we cannot always get at the artery at all, between the aneurism and the heart. This consideration led Dessault to *propose*, and it is said (by the younger Sprengel) to *perform*, with success, the ligature *beyond* the tumour. This operation is reported to have succeeded in aneurisms of the axillary and external iliac arteries, but we cannot find the proofs. DESCHAMPS followed the example of Dessault; but in the two operations which he performed for aneurism of the external iliac or inguinal

* De Efficaci Med. Lib. i. p. 11. p. 51.

artery, he completely failed. The same want of success attended Sir Astley Cooper, and it only remains to inquire, whether those performed by Mr. Wardrop, on the principle of Dessault, promise a more fortunate result. Of the four patients operated on by Mr. Wardrop and Mr. Lambert, two are dead, and in one of these, no aneurism, it has been shewn, was found, nor any trace of ligature having been applied to the carotid artery. The first case, the old lady, is still living, and dissection has not yet cleared up the true nature of the malady. Of the case operated on by Mr. Lambert, we gave our opinion in a former Number. From seeing the preparation and the drawing, we are still of opinion, that it was aneurism by *dilatation* of the carotid artery, a disease not necessarily fatal in itself. The patient died of hæmorrhage, from ulceration of the coats of the artery.

Of the last case operated on by Mr. Wardrop, we gave an account in our last Number, page 534. Dr. Barry's diagnosis of the disease may be seen in the same place, where he stated the innominata to be considerably *dilated*, as also the aorta within the pericardium and at its arch, where it pressed upon some of the bronchia. Dr. Barry also stated his opinion, that the left carotid was "uniformly-dilated." From these and other considerations, Dr. Barry pronounced the disease to be fatal in its results, and that no operation on the right carotid could be of any service. Nevertheless the subclavian was tied, and in the LANCET, we find the most flourishing accounts of the patient's subsequent health. She had lost her cough, her dyspnœa, all pain—and could go up stairs as well as ever. But Dr. Barry, in the Westminster Society, stated, from an accurate examination of the woman, that the dyspnœa, instead of being removed, was *increased* since the operation—and in short, there cannot be a doubt that, although the tumour or dilatation of the vessels was prevented from proceeding in an outward direction, it had been making progress in another and more dangerous direction, which would terminate ultimately (if it went on) by suffocation. Thus the patient had been relieved of *pain*, occasioned probably by the pressure of the aneurism on some nerve or nerves, but had got an increase of *dyspnœa*, which we consider a far more formidable symptom, and one which was likely to produce more suffering in the end.

From this exposé, we believe the Profession will be disposed to place little hope in the ligature *ultra tumorem*. When, indeed, an internal aneurism has protruded beyond the clavicle or the groin, there can be little rational hope of cure by any means. The cause of want of success, may fairly be attributed to the impossibility of arresting the current of blood through the aneurism, on which arrest the great hope of success must depend. Now the epigastric and circumflexa ilii arteries must always keep up a stream through an aneurism, where the ligature is applied beyond the tumour, in the groin or thigh—and the carotid, and several other arteries must also keep up the current in an aneurism of the innominata; so that the chance of success is diminished almost to a cipher. The only tangible artery, as we stated in a former Number, where ligature *ultra tumorem* offers any prospect of cure, is the carotid, because, from the common trunk of that vessel, no branch goes off till it divides at the top of the neck. In an aneurism, therefore, of that artery, situated too low for ligature *citra aneurismam*, there is a fair excuse for tying the vessel *ultra tumorem*. In any other situation, we fear the operation will be useless—perhaps, worse than useless.

P. S. Just as we had penned the above, Mr. Wardrop's additional report, dated the 5th December, came to our view. In this communication Mr. Wardrop impugns the statement made by Dr. Barry, above alluded to, and he tells us that *at the end* of August, Mrs. Denmark was so well that Mr. Chapman, who examined her, declared to Mr. Lawrence that, had he not been previously acquainted with the nature of the case, "he could never have discovered that aneurism of any of the *large vessels of the heart* had ever existed." *Lancet, Dec.* 8. Mr. W. further informs us that the patient's countenance had "lost entirely that anxious expressive look which was formerly so observable." And bringing up the account to the date of his report, (5th Dec.) says, "she *has* regained a nearly perfect state of health." The operator avers that, six weeks prior to the above date of report,

"not a vestige of the tumour remains"—"a perfect cure had been effected." Such was the statement on the 5th December. On the 12th December, we examined Mrs. Denmark with the greatest care. Her countenance was the very image and personification of distress and despair. Her breathing was extremely difficult—not the dyspnœa of inflamed lungs, but the difficulty of breathing through a compressed tube. The noise made by the effort to fill and empty the chest, was precisely that made by the rush of air through a flattened tube. Examination, by the stethoscope and pleximeter shewed the existence of the original aneurismal tumour, stretching from the heart towards the first rib. The pulse and impulse of the tumour were still unequivocal. It was perfectly evident that the tumour was pressing on the air-passages, and was thus the cause of the dreadful dyspnœa, as it would be, ultimately, of the death of the patient. Such was the state of this unfortunate woman's case, on the 12th December, four days after Mr. Wardrop's statement appeared before the public. We cannot reconcile the discrepancy of Mr. Wardrop's statement with the evidence of our own senses. We again saw this woman on the 16th December. She was then confined to her bed, and obliged to keep the trunk nearly perpendicular. The breathing was still more difficult than before—and a muco-purulent expectoration was brought up with difficulty from the lungs. On this, and on the former visit, we found the pulse in the right arm perfectly distinct, though not so strong as in the other arm. We have been informed, on the very best authority, that on the very day after the operation, and afterwards on the 10th or 12th day, consequently before the ligature came away, the pulse was distinctly felt by Mr. Lawrence and others, in the right arm. If this information be incorrect, Mr. Lawrence can easily contradict the statement, when we will give our authority, who also felt the pulse.—Was the artery tied—or, at least, was the canal obliterated?

On the 27th December, we again saw Mrs. D. Bleeding and leeching had occasionally relieved the difficulty of breathing; but the pressure of an aneurismal tumour on the trachea or bronchia was fearfully evident. At this time, we could not perceive the beat of the radial artery. The pulsation in the left carotid was tremendous. A kind of undulatory pulsation was perceptible in the situation of the original aneurism; but whether it was a *communicated* pulsation, or a motion of blood in the part, we could not distinctly ascertain. A muco-purulent expectoration was still thrown up—and when the expectoration accumulated, the difficulty of breathing was dreadful. The unfortunate patient could only repose in a nearly erect posture, and her sufferings were extremely distressing.

On Saturday, the 5th January, we had reason to know (though not from personal observation) that the aneurismal tumour was again taking an outward direction—that the clavicle was again rising, and being dislocated—and that, in short, the poor patient was nearly in the same pathological condition as before the operation of tying the subclavian artery—*ultra aneurismam!* This direction of the aneurismal tumour outwards, and the repeated bleeding which had been practised, had somewhat relieved the dyspnœa, and the muco-purulent expectoration was much lessened.

Our information respecting this poor woman comes down no later than the 14th January, when we understood that the clavicle was still rising—that the breathing was still very bad, though not so much so as at one or two periods within the last six weeks—and that no pulsation whatever could be felt in the right arm since the 27th December.

Now, as we can confidently state that the pulse was felt in the right wrist the day after the operation, and at all times up to the 27th December—and as all pulsation has now ceased in that arm, are we not authorised to *suspect* that the subclavian was never tied*—and are we not fully justified in asserting that, the cessation of pulsation in the arteries of the arm, of late, is solely owing to some pressure, or morbid *change* going on in the

* We are aware that it is not very uncommon for the pulse to return in an artery, after the Hunterian operation has been performed. This return, however, is only temporary; the pulsation is more or less indistinct, lasts for a few days, and disappears.

chest, and totally unconnected with the operation?

Upon the whole, a careful examination of all the cases now on record, including that of Mrs. Denmark, induces us to conclude that the ligature ultra aneurismam has failed, and that there are no facts to authorise a repetition of it. We conceive that we should not have been acting conscientiously to the profession, and to humanity, had we suppressed the true state of Mrs. Denmark's case, after a report had gone forth that she was perfectly cured.

### 2. ARM AND SHOULDER-PRESENTATIONS.

Dr. Robert Lee read a short paper before the Westminster Medical Society, on the 5th January, the object of which was to recommend the sacrifice of the child, in cases of arm and shoulder presentations, where turning was unadvisable or impracticable. He shewed that much mischief to the mother is sometimes done by attempts to turn, without saving the child after all. When, therefore, we have proof that the child is dead—that the pelvis is too small to allow a living child to pass —or, that there is great improbability of bringing a living child into the world, even by turning, we are advised to disjoint the arm at the shoulder, introduce a blunt hook, so as to take hold of the spine, as low down as possible—and bring away the fœtus double—thus imitating the operations of nature, in some cases of spontaneous evolution. Dr. Lee did not bring forward this procedure as entirely new, since Dr. Douglas and some others had proposed it. He related three cases in illustration. Some comments were made by Mr. Jewel, as to the novelty of the proposal, but no serious objections made to the practice.

### 3. PURULENT DEPÔTS, AFTER WOUNDS AND OPERATIONS.

Mr. Rose, of St. George's Hospital, read a paper to the Medico-Chirurgical Society, on the 7th January, on the above subject. It has been observed by various foreign and English writers, that abscesses (as they were called) occasionally took place in the liver and lungs, especially in the former viscus, after wounds, operations, and accidents. According to continental experience, the liver is the organ most commonly affected; but, in English practice, the lungs are full as often the scene of the purulent deposition. The peculiarity is, that the matter, if it be entitled to that name, is collected in one or more globular depôts, not at all like common abscesses of the parts, the surrounding structure being generally sound. The deposition itself is a mixture of coagulable lymph and puriform striæ. Mr. Lawrence related a case, where the surface, and even the substance of the liver, were found studded with some hundreds of these globular depôts, after an operation on the knee-joint. A warm discussion took place between Dr. Seymour, Mr. Lloyd, and Dr. Johnson, as to the frequency or infrequency of common circumscribed abscess (unconnected with tubercles) in the parenchymatous structure of the lungs, in acute inflammation. According to Dr. Seymour, the occurrence was by no means uncommon—according to Mr. Lloyd, one would suppose that, *nothing was more common*—according to Dr. Johnson, it was extremely uncommon—so much so, that he had never met with a well-marked instance of it, as proved by dissection.* The authority of Laennec was opposed by that of Dr. Luke! This put an end to the discussion. As Mr. Rose's paper will soon be published, we shall abstain from any remarks at present; but we promise to meet Messrs. Seymour and Lloyd before a larger audience, on the above-mentioned subject, ere long.

### 4. AUSCULTATION.

In two recent sittings of the Westminster Medical Society, the important subjects of auscultation and percussion were warmly discussed; and never was triumph more complete than that obtained by the advocates of these modern means of ascertaining thoracic diseases! In compassion to the opposers of these improvements in medical science, we shall draw a veil over their names. There were only *three*,

* Mr. Lawrence only remembered one example, and that appeared such a rarity, that he preserved the part in his museum.

in a society consisting of nearly 100 members and visitors. On the first night, there was a diarrhœa, we might say a *lientery* of declamation poured forth against auscultation and percussion, without a single fact or argument being adduced, that was worthy of refutation. Two facts *only* were brought forward, and these we shall state, without deigning to honour them with a comment. The first fact occurred before auscultation was born. A young woman was admitted into the Royal Infirmary of Edinburgh, some 20 or 30 years ago, with swellings in both knees. The surgeons, anxious to shew their dexterity, amputated one of the lower extremities; and, on examining the joint, found there was no organic disease to authorise the operation. They then set their brains to work, and cured the disease in the other knee, without amputation:—ergo, AUSCULTATION was a most pernicious measure introduced into medical diagnosis! As our country readers might not believe that such a fact could be seriously presented to a medical society, as an objection to auscultation, we shall explain a little. The gentleman who introduced this *case*, in the way of illustration, observed that, as the Edinburgh surgeons were more intent on shewing their dexterity with the knife, than their therapeutical knowledge in the cure of the swelling of the knee, so the auscultators would neglect all investigations, except by the stethoscope, and thus become non-observers of the phenomena of thoracic diseases!!

*Fact the Second.* Two patients died in the Middlesex Hospital, on one of whom, a student had been making some stethoscopical observations. On repairing to the dead-house, the young pupil began to examine the *wrong* body, through a mistake—*ergo*, the dreadful injuries which must result to medical science from this zeal which some young and raw students evince in opening bodies (wrong bodies) after death!

These were the *only facts* brought into court against auscultation and percussion; and the anti-auscultators candidly acknowledged, that they had never made the least attempt to examine into the value of the diagnostic measure! They poured forth an abundant declamation against the stethoscope, as a "foreign bauble," an "inutile lignum"—an "insult to John Bull"—and, in short, a piece of "continental quackery." They had (like Mr. Lawrence) the *last words*, in the evening of the first night. But a terrible humiliation was in reserve for them in the second night's discussion. On this night two out of the three *ratted*, and passed the highest eulogia on auscultation; while the third was completely nonplused, and merely *entered his protest* against auscultation, without adducing a single fact or cogent argument against the measure.

On the other hand, the value and utility of this auxiliary to diagnosis was ably maintained by Mr. Mackelcan, Dr. Barry, Dr. Shiel, Mr. Bennett, Dr. Milligan, Dr. Johnson, and others. Dr. Barry, in a luminous speech, shewed the wretched state in which the pathology of the chest was, before the days of Avenbrugger, Corvisart, and Laennec. It is true that Dr. Baillie has left us a description of diseased appearances, which is fit for nothing, except a catalogue of a museum. Dr. B. described the morbid changes *before* he saw the symptoms—and when he described the symptoms, he had no opportunity of seeing the morbid changes. Thus, the connexion between symptoms and changes of structure, in Dr. Baillie's work, is purely *imaginary*. Yet, on this connexion, the whole value of pathology depends. Hence the great merit of Laennec and other modern pathologists consists in demonstrating the external signs of internal morbid processes.

Among other futile and erroneous objections that were made to auscultation, it was urged that, when diseases were so far advanced as to be recognizable by the stethoscope, they were incurable—ergo, auscultation was useless. A damper was thrown on this line of argument by the case of a man, residing within 500 yards of the Society's rooms, and who had been seen by three physicians then present in the Society. This man had been in two public institutions—in one he was treated for nervous asthma—and ordered to take beef-steaks and porter. There he got worse; so he went to another institution, where he was treated for phthisis—and there he got no better. Dr. Johnson was requested to see this poor man, and, on examining the chest, an aneurism was discovered, without the least difficulty, beating under the right clavicle. The case was shewn to Dr. Barry and another physician, who were appealed to for the accuracy of the statement. The man was bled, put upon low diet, and his breathing was, by these means, much relieved. Here, then, was an instance of the *injurious* consequences of auscultation. Dr. Barry related two or three recent cases, where the most erroneous views of disease had been taken, and where an injurious mode of treatment was being pursued, till auscultation set the practitioners on the proper road. In fine, the the anti-auscultators were reduced to a single theme of declamation--namely, that the stethoscope would render the rising generation of the profession less attentive observers than their fathers and grandfathers. We have only to say, that it will have just the opposite effect. It will make them much better observers.

---

### LIGATURE OF THE COMMON ILIAC ARTERY. DR. V. MOTT.

This operation, which throws into the shade every other of the kind, yet placed on record, has recently been performed, *with success*, by one of our transatlantic brethren. We shall proceed, at once, to detail this brilliant triumph of modern surgery.

On the 15th March, 1827, Dr. Mott was called, with Dr. Osborn, to a patient, who was found labouring under a large aneurism of the right external iliac artery. The man's name was Israel Crane, aged 33 years, a farmer, of temperate habits, but accustomed to hard and laborious work. About the middle of January he felt pain in the lower part of the belly, which he attributed to a fall formerly received. It was only a fortnight previously, however, that he perceived the tumour. On examination, the abdomen, on the right side, was considerably enlarged, from the crural arch to the umbilicus, the swelling being pulsatile, both to the touch and the sight. It appeared to contain fluid blood only. It commenced a little above Poupart's ligament, reached nearly to the navel, advanced forward near the linea alba, and seemed to fill up all the concavity of the ilium.

"The rapid increase of this aneurismal tumour occasioned, as the countenance of our patient indicated, the most extreme agony. His sufferings at times were so great that his screams could be heard at a distance from the house. He had been bled several times, taken light food, and was kept constantly under the effect of opium. He was now informed of the serious nature of his case, and that without an operation, very little chance of his life remained; with great composure he immediately consented to whatever would give him the best prospect of saving his life.

"From the extent and situation of the tumour, he was apprised of the uncertain nature of the operation, as well as the difficulty of performing it, and indeed that it would require an artery to be tied, which never had been before operated upon for aneurism. With these views of his situation, he cheerfully submitted to be placed upon a table of suitable height in a room which was well lighted.

"Then, in the presence of Dr. OSBORN, Dr. LIDDLE, and Dr. CROSS, the following operation was performed:—

"The pubes and groin of the right side being shaved, an incision was commenced just above the external abdominal ring, and carried, in a semicircular direction, half an inch above Poupart's ligament, until it terminated a little beyond the anterior spinous process of the ilium, making it in extent about five inches The integuments and superficial fascia were now divided, which exposed the tendinous part of the external oblique muscle, upon cutting which in the whole course of the incision, the muscular fibres of the internal oblique were exposed; the fibres of which were cautiously raised with the forceps and cut from the upper edge of Poupart's ligament. This exposed the spermatic cord, the cellular covering of which was now raised with the forceps, and divided to an extent sufficient to admit the forefinger of the left hand to pass upon the cord into the internal abdominal ring. The finger serving now as a director, enabled me to divide the internal oblique and transversalis muscles to the extent of the external incision, while it protected the peritoneum. In the division of the last mentioned muscles outwardly, the circumflexa ilii artery was cut through, and it yielded for a few minutes a smart bleeding. This, with a smaller artery upon the surface of the internal oblique muscle, between the rings, and one in the integuments were all that required ligatures.

"With the tumour beating furiously underneath, I now attempted to raise the peritoneum from it, which we found difficult and dangerous, as it was adherent to it in every direction. By degrees we separated it with great caution from the aneurismal tumour, which had now bulged up very much into the incision. But we soon found that the external incision did not enable us to arrive to more than half the extent of the tumour upwards. It was therefore extended upwards and backwards about half an inch within the ilium, to the distance of three inches, making a wound in all about eight inches in length.

"The separation of the peritoneum was now continued, until the fingers arrived at the upper part of the tumour, which was found to terminate at the going off of the internal iliac artery. The common iliac was next examined by passing the fingers upon the promontory of the sacrum, and to the touch appearing to be sound, we determined to place our ligature upon it about half way between the aneurism and the aorta, with a view to allow length of vessel enough on each side of it to be united by the adhesive process.

"The great current of blood through the aorta made it necessary to allow as much of the primitive iliac to remain between it and the ligature as possible, and the probable disease of the artery higher than the aneurism, required that it should not be too low down. The depth of this wound, the size of the aneurism, and the pressure of the intestines downwards by the efforts to bear pain, made it almost impossible to see the vessel we wished to tie. By the aid of curved spatulas, such as I used in my operation upon the *innominata*, together with a thin smooth piece of board, about three inches wide, prepared at the time, we succeeded in keeping up the peritoneal mass, and getting a distinct view of the arteria iliaca communis, on the side of the sacro vertebral promontory. This required great effort on our part, and could only be continued for a few seconds. The difficulty was greatly augmented by the elevation of the aneurismal tumour, and the interception it gave to the admission of light.

"When we elevated the pelvis, the tumour obstructed our sight; when we depressed it, the crowding down of the intestines presented another difficulty. In this part of the operation I was greatly assisted by Dr. Osborn, and my enterprising pupil, ADRIAN A. KISSAM.

"Introducing my right hand now behind the peritoneum, the artery was denuded with the nail of the forefinger, and the needle conveying the ligature was introduced from within outwards, guided by the forefinger of the left hand in order to avoid injuring the vein. The ligature was very readily passed underneath the artery, but considerable difficulty was experienced in hooking the eye of the needle, from the great depth of the wound and the impossibility of seeing it. The distance of the artery from the wound was the whole length of my aneurismal needle.

"After drawing the ligature under the artery, we succeeded by the aid of our spatulas and board in getting a fair view of it, and were satisfied that it was fairly under the primitive iliac, a little below the bifurcation of the aorta. It was now tied—the knots were readily conveyed up to the artery by the forefingers—all pulsation in the tumour instantly ceased. The ligature upon the artery was very little below a point opposite the umbilicus.

"The wound was now dressed with five interrupted sutures, passing them not only through the integuments, but the fibres of the cut muscles, so as to bring their divided edges together at all parts of the incision, which was muscular. Adhesive plaster to assist the stitches, lint and straps to retain it, completed the dressing. The operation lasted rather less than one hour."

The space which we have dedicated to the steps of the operation, prevents us from giving a detail of the diurnal reports, carefully drawn up by Dr. Osborn, and authenticated in the most satisfactory manner. There was very little constitutional disturbance produced by this formidable operation. The temperature of the limb was soon restored:—The patient was bled a few times—kept quiet and low. The operation was performed on the 15th March, and on the 3rd of April Dr. Proudfoot removed the large ligature, the wound being almost healed. On the

16th the patient imprudently rode out, without permission, the wound being perfectly healed. On the 30th April, he was in complete health. The leg was not of its full size, nor so strong as the other—he was free from pain. On the 20th May, he came 25 miles to shew himself in New York. The remains of the aneurismal tumour had disappeared—the epigastric artery was found much enlarged, and beating strongly, and there was a feeble pulsation in the femoral artery.

When we compare this operation with the one performed some months ago by Mr. Wardrop, and reflect that in a case like Crane's, the operation *ultra aneurismam* would have been applicable, according to the doctrines recently maintained, we must admit that the interests of surgery and of humanity demanded a full and speedy exposition of the newly revived doctrine of Dessault, and the data on which it is grounded. The facts which we have here put upon record speak for themselves; and we shall not add a single comment.

